# Electronic Two-Transition-Induced Enhancement of Emission Efficiency in Polymer Light-Emitting Diodes

**DOI:** 10.3390/ma6030886

**Published:** 2013-03-06

**Authors:** Ren-Ai Chen, Cong Wang, Sheng Li, Thomas F. George

**Affiliations:** 1 Department of Physics, Zhejiang Normal University, Jinhua, Zhejiang 321004, China; E-Mails: cra_wz_2008@163.com (R.-A.C.); wangimagine@gmail.com (C.W.); 2 Department of Physics, Fudan University, Shanghai 321004, China; 3 Office of the Chancellor and Center for Nanoscience, Departments of Chemistry & Biochemistry and Physics & Astronomy, University of Missouri—St. Louis, St. Louis, MO 63121, USA

**Keywords:** light-emitting diodes, polymers, excitons

## Abstract

With the development of experimental techniques, effective injection and transportation of electrons is proven as a way to obtain polymer light-emitting diodes (PLEDs) with high quantum efficiency. This paper reveals a valid mechanism for the enhancement of quantum efficiency in PLEDs. When an external electric field is applied, the interaction between a negative polaron and triplet exciton leads to an electronic two-transition process, which induces the exciton to emit light and thus improve the emission efficiency of PLEDs.

## 1. Introduction

Because of the potential advantages (flexibility, full color capability, low cost, ease of fabrication, *etc.*) of organic light-emitting diodes (OLEDs) or polymer light-emitting diodes (PLEDs) as optoelectronic devices [1,2,3], they have become “hot” research topics during the last several decades. According to quantum statistics and the Pauli Exclusion Principle, the maximum quantum efficiency of the PLEDs/OLEDs is limited to 25%, which is due to the ratio of the radiative singlet exciton to the non-radiative triplet exciton being 1:3. The simplest structure of OLEDs/PLEDs is, generally, like a sandwich—an emitting layer between an anode and cathode. In order to improve the quantum efficiency, a hole injection layer (HIL) and electron injection layer (EIL) are embedded in the OLEDs/PLEDs.

However, the poor transport of an electron injection layer leads to an imbalance between the hole and electron currents, thus lowering the device performance. Further, as low-work-function metal cathodes for electron injection, such as Li, Ca and Mg, are not stable in air, it becomes necessary to rigorously encapsulate the devices. Thus, emissive polymeric materials tend to be based on hole injection and transport [4,5,6].

In order to improve the efficiency and stability of electron-injected OLEDs/PLEDs, it has been found that once a hole-blocking layer is embedded into the extremely flexible liquid-emitting OLED layer, the injected electron improves the quantum efficiency of the device to 55% [7]. Cao *et al.* have reported that electroluminescence (EL) is greatly enhanced after blending electron transport materials with the conjugated polymer [8]. It is also found that if an electron injection layer (EIL) is embedded in an OLED, the electroluminescence efficiency significantly increases by about two orders of magnitude compared to that of a device without an EIL [9]. To avoid the instability of metallic materials with EIL, Friend *et al.* have used metal oxide polymers as EILs in PLEDs [10]. In addition, titanium and titanium dioxide have become excellent choices as EILs in PLEDs [11].

After modifying EILs and electron transport layers in OLEDs/PLEDs, it has been discovered, near the surface or heterojunction of different organic layers, that the formed exciplex, namely, one molecule (electron donor) in the excited state coupled with the other (electron acceptor) in the ground state, is able to enhance the fluorescent efficiency of OLEDs/PLEDs [12,13,14,15]. Recently, Park *et al.* demonstrated that an EIL can strengthen the above effect leading to the improvement of the EL efficiency [16]. Nevertheless, considering the weak binding energy of the exciplex, the thermal excitation could drive the exciplex away from the heterojunction to the bulk of the polymer. Inside the bulk of the polymer, the exciplex can easily transform to an exciton [17]. Therefore, despite the above surface effect, there probably exists other channels inside the polymer, which enables the exciton to emit light effectively.

In 2007, an EDMR (electrically detected magnetic resonance) experiment showed that it is highly possible for trapped electrons to be negative polarons. As a result of the interaction between a negative polaron and hole, a new exciton can be formed, leading to radiative decay [18]. Based on this, it is assumed that the injection of electrons, apart from balancing the charge carriers, enables the generated negative polarons to interact with the excitons, thus changing the light efficiency. Hence, in this paper we will focus on whether and how the dynamic process of electron injection affects the exciton emission of the polymer.

The theory concerning electronic factors in energy transfer and molecular exciton interactions has been under development for more than ten years [19,20]. The Pariser–Parr–Pople method, time-dependent density functional theory, and sophisticated *ab initio* calculations have also been employed to study singlet and triplet exciton energy structures and dynamics in electronic materials and devices [21,22,23]. These have already revealed a great proportion of the underlying properties of quasi-particles in organic semiconductors. Nevertheless, our band-theory picture, as the Su–Schrieffer–Heeger model [24] represents, is another appropriate and powerful approach for our system in describing transportation dynamics and energy structure in conjugated polymers. Our previous work has looked at photoinduced carrier fission [25], field-induced spin accumulation in PLEDs [26], forbidden singlet transitions in a strong electric field [27], and dipole moment related singlet exciton decay [28]. Here, we will apply our established technique of transitional molecular dynamics to the unique quasi-one-dimensional structure of conjugated polymers in order to unveil the new channel causing high efficient fluorescent PLEDs and to illustrate the dynamic fluorescence spectra of the whole dynamic process.

## 2. Method

When the electron-electron interaction is included, the extended Su–Schreiffer–Heeger–Hubbard model [24] becomes a powerful tool for quantitatively describing the properties of the conjugated polymer. In addition, for the confinement effect of a nondegenerate polymer, the Brazovskii–Kirova symmetry-breaking term [29] is added to this model. The resulting Hamiltonian is

(1) $$H=-\sum_{l,s}[t_{0}+\alpha (u_{l+1}-u_{l})+{\left(-1\right)}^{l}t_{e}]\times [{c^{+}}_{l+1,s}c_{l,s}+H.c.]+\frac{K}{2}\sum_{l}{\left(u_{l+1}-u_{l}\right)}^{2}+H_{e}+H_{E}$$

(2) $$H_{e}=U\sum_{l}n_{l,↑}n_{l,↓}+V\sum_{l,s,s^{\prime }}n_{l,s}n_{l+1,s^{\prime }}$$

(3) $$H_{E}=\sum_{l,s}Ee(l-\frac{N+1}{2})an_{l,s}$$

The parameters here are specified as follows: $t_{0}$ is a hopping constant (2.5–5.0 eV); $t_{e}$ is the Brazovskii-Kirova term [29] (0.05–0.10 eV); *α* is an electron-lattice coupling constant (4.3–5.6 eV/Å); ${c^{+}}_{l,s}(c_{l,s})$ denotes the electron creation (annihilation) operator at cluster l with spin s, $u_{l}$ is the displacement of cluster l, and *K* is an elastic constant (19–24 eV/Å^2^); *U* (2.0–5.0 eV) and *V* (0.5–2.0 eV) are the on-site and nearest-neighbor Coulumb interactions, respectively, a (1.2–3.8 Å) the lattice constant, *H*_E_ is the interaction of the electrons with the external electric field along the polymer chain, *E* (1.0 × 10^2^–5.0 × 10^3^ V/cm) is the electric field strength; and *N* is the number of lattice site.

The electron-electron interaction term *H*_e_ can be treated by the Hartree–Fock approximation [25,26], and thus the eigenvalue equation of the above Hamiltonian, $H\Phi_{\mu }=\varepsilon_{\mu }\Phi_{\mu },$ takes the form

(4) $$\varepsilon_{\mu }Z_{l,\mu }^{s}=[U\left(\rho_{l}^{-s}-\frac{1}{2}\right)+V(\sum_{s^{\prime }}\rho_{l-1}^{s^{\prime }}+\sum_{s^{\prime }}\rho_{l+1}^{s^{\prime }}-2)+Ee\left(l-\frac{N+1}{2}\right)a]Z_{l,\mu }^{s}\;-[V\sum_{\mu }^{occ}Z_{l,\mu }^{s}Z_{l-1,\mu }^{s}+t_{0}+\alpha (u_{l-1}-u_{l})+{\left(-1\right)}^{l-1}t_{e}]Z_{l-1,\mu }^{s}\;-[V\sum_{\mu }^{occ}Z_{l,\mu }^{s}Z_{l+1,\mu }^{s}+t_{0}+\alpha (u_{l+1}-u_{l})+{\left(-1\right)}^{l+1}t_{e}]Z_{l+1,\mu }^{s}$$

where the wavefunction $\Phi_{\mu }=\{Z_{l,\mu }^{s}\}$ and charge distribution are defined as $\rho_{l}^{s}=\sum_{\mu }^{occ}|Z_{l,\mu }^{s}{|}^{2}-n_{0}$ ($n_{0}$ is the density of the positively-charged background).

Since atoms are much heavier than electrons, based on the Feynman-Hellmann theorem, an atom’s movement can be described by classical dynamics as

(5) $$M\frac{d^{2}u_{l}}{dt^{2}}=-\sum_{\mu }^{occ}\frac{\partial \varepsilon_{\mu }}{\partial u_{l}}+K(2u_{l}-u_{l+1}-u_{l-1})$$

These coupled equations can quantitatively describe the dynamics of a conjugated polymer chain. In order to further depict the process of electronic transitions between different levels, the electron population rate equations are introduced [27,28]. If there are three energy levels marked by *a*, *b* and *c*, the evolutions of their related electron populations, $P_{a},$ $P_{b},$ and $P_{c},$ are presented as

(6) $$\frac{dP_{a}}{dt}=-\gamma_{ab}P_{a}\;\frac{dP_{b}}{dt}=\gamma_{ab}P_{a}-\gamma_{bc}P_{b}\;P_{c}=n-P_{a}-P_{b}$$

Where $\gamma_{ab}(\gamma_{bc})$ is the transition rate between energy levels *a* and *b* (*b* and *c*), and *n* is the total electron number. Using the above equations and molecular dynamics, the whole two-transition process in electroluminescence is demonstrated.

## 3. Calculations and Results

**Figure 1:**
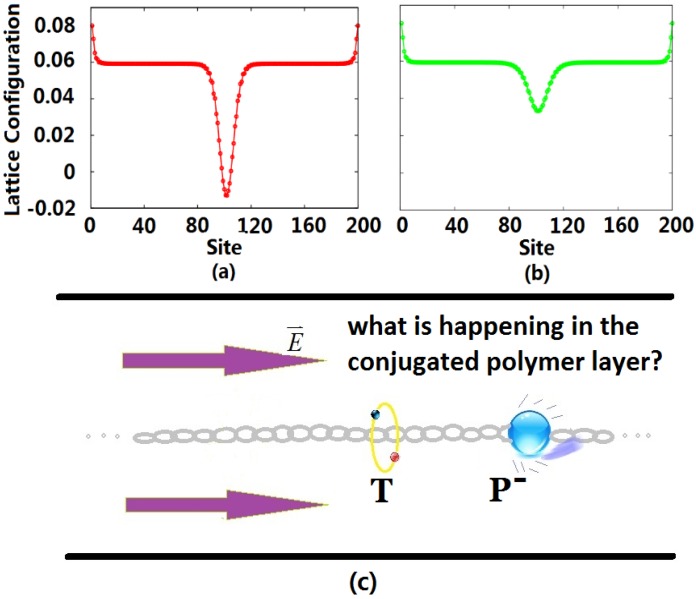
Lattice configuration of the triplet exciton (**a**) and negative polaron (**b**), where the unit of the vertical axis is Angstroms; and schematic graphic (**c**) of the collision of the negative polaron (P) and triplet exciton (T) inside the conjugated polymer layer. Initially, the triplet exciton stays in the middle of the polymer chain, and the polaron starts from the right side of the chain.

After the exciplex escapes from the heterojunction into the bulk of the polymer due to thermal excitation, the initial homogeneous dimerization lattice configuration of the conjugated polymer is no longer stable, such that it undergoes localized distortion to form a self-trapping exciton. At this time, the singlet exciton begins to transit radiatively with a lifetime of about 1 ns, but because of the limitation of the Pauli exclusion principle, the triplet exciton cannot radiatively decay. For electron injection in OLEDs, the excess electrons entering the LUMO also distort the lattice, forming a negative charge-carrier polaron, which is the ground state that can exist stably in the polymer. Defining the lattice configuration through an order parameter, we display the configurations for the triplet exciton and negative polaron in Figure 1. Due to the electric field, the negative polaron moves in the direction against the polymer chain towards the triplet exciton localized in the middle of the chain, as shown in Figure 1c.

**Figure 2:**
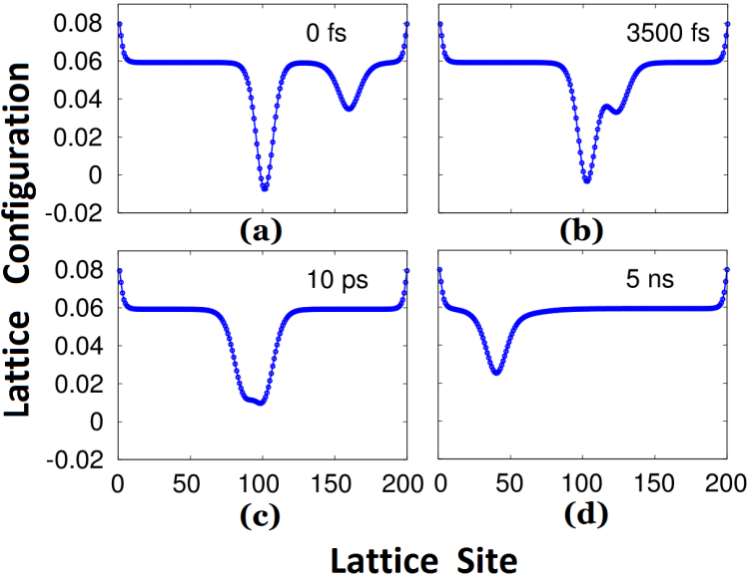
Evolution of the lattice configuration during the process of the interaction between the negative polaron and triplet exciton under an external electric field.

As the negative polaron approaches the triplet exciton, the evolution of the lattice configuration is shown in Figure 2. Up to 3500 fs, as illustrated by Figure 2b, the polaron interacts with the exciton, and simultaneously the polaron continues to move (slowly) to the left due to the external electric field. When the time reaches 10 ps, the lattice configure shows these two carriers fusing together. At the time of 5 ns, the lattice configuration distortion becomes not only “narrower” but also “smaller”, as depicted in Figure 2d, which is similar to the polaron in Figure 1b but localized on the left of the polymer chain.

**Figure 3:**
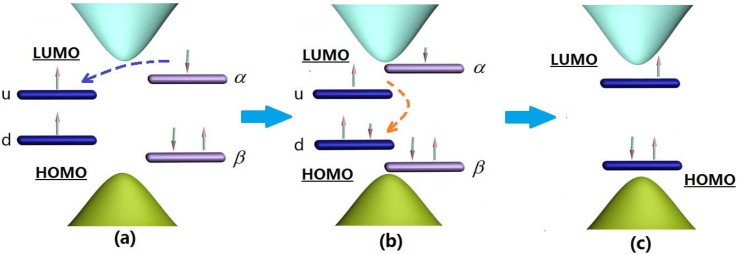
Schematic diagram of the electron population during the electronic two-transition process.

Figure 3 depicts a physical picture of the whole process. When the negative polaron approaches and begins to couple with triplet exciton, their own localized states start to overlap. Though the electron in energy level *u* of the exciton cannot transit from energy level *d*, *u* can borrow “empty room” for the electron of the polaron, with opposite spin in energy level *α*, and transit from *α* to *u*, as marked with a blue arrow in Figure 3a. Then the electron continues to transit from *u* to *d* of the exciton, as marked by the red arrow in Figure 3b, to finally achieve the electronic two-transition process.

**Figure 4:**
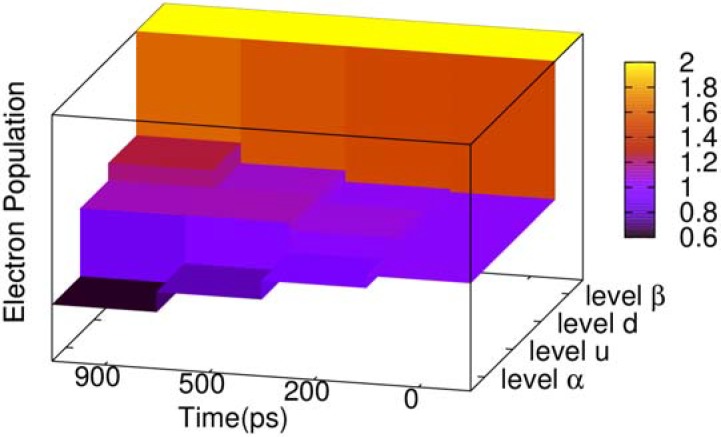
Three dimensional (3D) column chart of the evolution of the electron population of four energy levels of the negative polaron and triplet exciton for the time points of 0, 200, 500 and 900 ps.

During the two-transition process, the electron population of the four related energy levels in the center of gap also changes, as depicted in Figure 4. The energy level *β* (see Figure 3) is always fully occupied by electrons, and its electron population never changes. Energy level *α* keeps decreasing, where the population remains 0.63 when time reaches 900 ps. To the contrary, energy level *d* is increasing, and its electron population grows to 1.24 when time reaches 900 ps. However, we see that the electron population of energy level *u* does not increase monotonously, where the electron of energy level *u* increases to 1.124 since the beginning, but at 700 ps begins to decrease. When time reaches 900 ps, the populations become 1.121. The reason is that at the beginning of the two-transition process, the electron population in *u*, with spin down, is zero. On the other hand, the population in energy level *α* is 1, such that it is available for most electrons with spin down to transit from *α* to *u*, but with few electrons from *u* to *d*. After energy level *u* accumulates a certain number of electrons with spin down, the population in *d* due to the transition from *u* starts to grow, and the population in *u* is gradually reduced.

In fact, during the electronic two-transition process, the interaction between the triplet exciton and negative polaron fuses them, with radiative decay as a single quasi-particle. At the end of the process, the energy spectrum is no longer an exciton, but a negative polaron (Figure 3c), and finally radiating photons.

**Figure 5:**
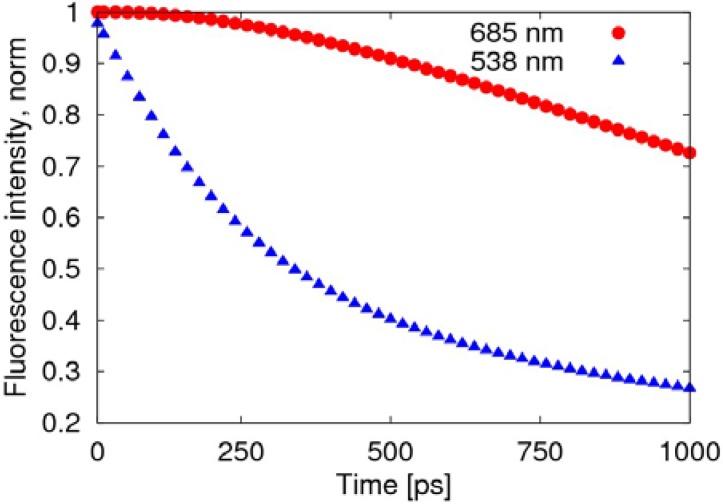
Time evolution of the fluorescence spectra after normalization. The red round points (685 nm) represent light emitted by the transformation of the triplet exciton, while the blue triangular points (538 nm) represent the light emitted by the decay of the singlet exciton.

The radiative transition, due to the fusion of negative polaron and triplet exciton, is much slower than the decay of the singlet exciton, as shown in Figure 5, where the fluorescence intensity deceases to 70% after about 1 ns, while the decay of the exciton remains at 27%.

**Figure 6:**
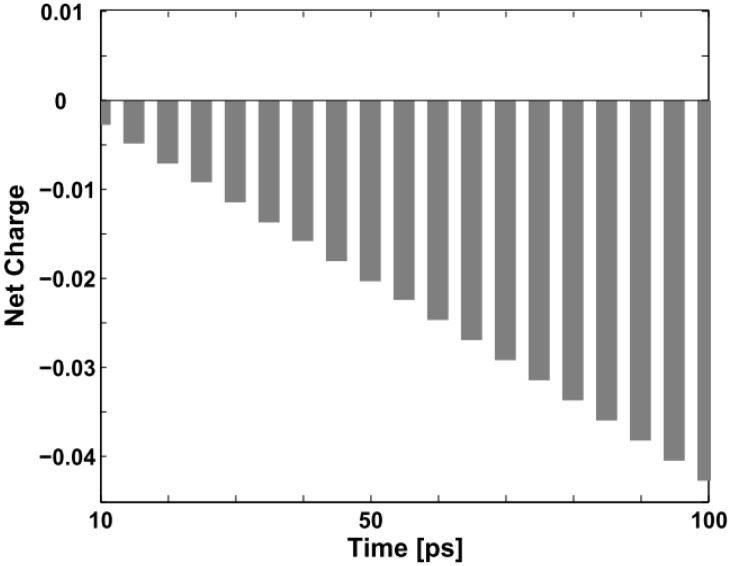
Net charge variation of the triplet exciton within the first 100 ps (the unit on the vertical axis is +|e|).

One should pay special attention to the collision process in order not to be misled by the concept of triplet exciton quenching by trapped and free charges in certain experiments. Although the original neutral triplet exciton no longer exists, it does not disappear *per se*. In fact, it combines with the negative polaron and transforms into another conformation. As indicated in Figure 2 and Figure 3, we may aptly call this new confinement state a “charged exciton” or “partially neutralized polaron”, a kind of quasi-particle, which also has been predicted theoretically and observed experimentally in electronically-doped OLEDs [30,31]. Obviously, it is not the triplet exciton itself nor a polaron, but this “charged exciton” emits light and enhances the quantum efficiency of the devices. Figure 6 below depicts the evolution of the net charge of the triplet exciton from “neutral” to “charged” during the first 100 ps of the collision with the negative polaron. 

Generally, the electroluminescent quantum efficiency is described by the formula

(7) $$EQE=\eta_{capture}\times \eta_{spin}\times \eta_{rad}\times \eta_{escape}$$

Here, $\eta_{capture}$ denotes the component of recombination, $\eta_{spin}$ is a factor of spin statistics ($\eta_{spin}$ = 1/4 for a singlet fluorescent material), $\eta_{rad}$ represents the component of formed radiative excitons (in ideal conditions, $\eta_{rad}$ ~ 100% ), and $\eta_{escape}$ is the ratio of escaped photons from the surface of the device to the photons formed by the recombination of excitons. Thus, without considering specific devices, we have the internal quantum efficiency:

(8) $$\eta_{\text{int}ernal}=\eta_{capture}\times \eta_{spin}\times \eta_{rad}$$

If we assume $N_{S}$ to be the number of singlet exciton and $N_{T}$ the number of triplet exciton which can transit radiatively by transformation, we then get

(9) $$\eta_{spin}\propto (N_{S}+N_{T})$$

**Figure 7:**
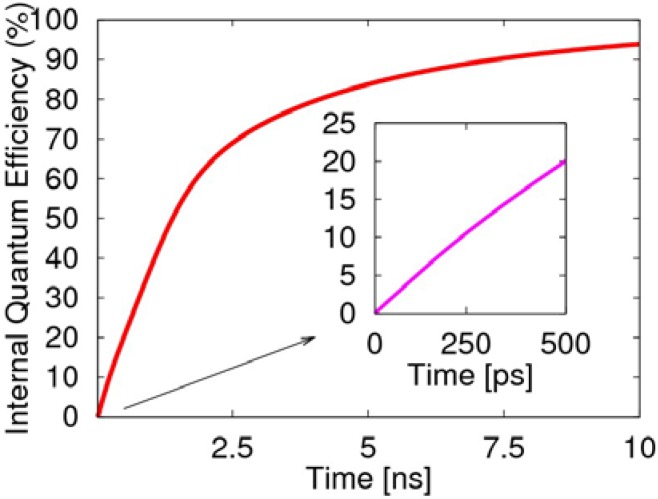
Evolution of the electroluminescent internal quantum efficiency, including the decay of the singlet exciton and emission from the two-transition process of the triplet exciton.

At the beginning in 0.5 ns of the two-transition process, the internal quantum efficiency rises rapidly to 20%, as illustrated in Figure 7, which is mainly due to the radiative decay of the singlet exciton. Afterwards, the two-transition process of the triplet exciton becomes the main factor for the light emission. If we consider the spins as randomly distributed and the injected electrons as sufficient enough, then the two-transition process will effectively cause the radiative transition of the triplet exciton to produce negative polarons. This also demonstrates the two-transition process not only induces the non-emissive triplet exciton to emit light, but removes the limitation of 25% for the quantum efficiency of the PLED, and the quantum efficiency even exceeds 80%. Clearly, the transient singlet excitons are the main contribution to the first 500 ps, while the long-lived triplet excitons dominate the later growth of the efficiency.

## 4. Conclusions

In summary, after the electron injection layer or electron transport layer is embedded into a PLED, the electrons are captured to form negative polarons. Driven by an external electric field, they fuse with triplet excitons in the bulk of the polymer. A two-transition process during the interaction between them not only induces the non-emissive triplet exciton to emit light, but removes the limitation of 25% for the quantum efficiency of the PLED, which even exceeds 80%. In addition, the whole process of transition dynamics is demonstrated, including the time evolution of the electron population and the details of electron transfer and dynamic fluorescence spectra, which should provide a new pathway for the enhancement of the emission efficiency of PLEDs. Although many factors that can influence the construction of LEDs, we suggest that the insertion of effective electron injection or transporting layers and the better balance of the carriers of both electrons and holes will surely raise the efficiency of LEDs from a practical perspective.
